# Neurofascin antibodies in autoimmune, genetic, and idiopathic neuropathies

**DOI:** 10.1212/WNL.0000000000004773

**Published:** 2018-01-02

**Authors:** Elisabeth Burnor, Li Yang, Hao Zhou, Kristina R. Patterson, Colin Quinn, Mary M. Reilly, Alexander M. Rossor, Steven S. Scherer, Eric Lancaster

**Affiliations:** From the Department of Neurology (E.B., K.R.P., C.Q., S.S.S., E.L.), University of Pennsylvania, Philadelphia; Department of Neurology (L.Y., H.Z.), Second Xiangya Hospital of Central South University, Changsha, China; and MRC Centre for Neuromuscular Diseases (M.M.R., A.M.R.), UCL Institute of Neurology and National Hospital for Neurology and Neurosurgery, London, UK.

## Abstract

**Objective:**

To measure the frequency, persistence, isoform specificity, and clinical correlates of neurofascin antibodies in patients with peripheral neuropathies.

**Methods:**

We studied cohorts of patients with Guillain-Barre syndrome (GBS) or chronic inflammatory demyelinating polyneuropathy (CIDP) (n = 59), genetic neuropathy (n = 111), and idiopathic neuropathy (n = 43) for immunoglobulin (Ig) G and IgM responses to 3 neurofascin (NF) isoforms (NF140, NF155, and NF186) using cell-based assays.

**Results:**

Neurofascin antibodies were more common in patients with GBS/CIDP (14%, 8 of 59) compared to genetic neuropathy controls (3%, 3 of 111, *p* = 0.01). Seven percent (3 of 43) of patients with idiopathic neuropathy also had neurofascin antibodies. NF155 IgG4 antibodies were associated with CIDP refractory to IV immunoglobulin but responsive to rituximab, and some of these patients had an acute onset resembling GBS. NF186 IgG and IgM to either isoform were less specific. A severe form of CIDP, approaching a locked-in state, was seen in a patient with antibodies recognizing all 3 neurofascin isoforms.

**Conclusions:**

Neurofascin antibodies were 4 times more frequent in autoimmune neuropathy samples compared to genetic neuropathy controls. Persistent IgG4 responses to NF155 correlated with severe CIDP resistant to usual treatments but responsive to rituximab. IgG4 antibodies against the common domains shared by glial and axonal isoforms may portend a particularly severe but treatable neuropathy. The prognostic implications of neurofascin antibodies in a subset of idiopathic neuropathy patients and transient IgM responses in GBS require further investigation.

Guillain-Barre syndrome (GBS) and chronic inflammatory demyelinating polyneuropathy (CIDP) are the 2 most common forms of autoimmune neuropathy. Both diseases are highly variable in the degree that sensory and motor axons are affected, the degree of demyelination and axonal injury, and the response to treatments. The immunologic basis of this variability is poorly understood.

Autoantibodies against membrane proteins expressed on axons or myelinating Schwann cells have recently been reported in some patients with CIDP or GBS. The target antigens are concentrated at nodes of Ranvier or at paranodes and are often cell adhesion molecules (CAMs) such as NrCAM, gliomedin, contactin-1, neurofascin (NF) 186, and NF155.^1–7^ Immunoglobulin (Ig) G4 antibodies to NF155 or its binding partner, contactin, have been associated with severe CIDP resistant to IV immunoglobulin (IVIG) treatment but potentially responsive to rituximab.^2,7,8^

We do not yet know the full utility of neurofascin antibodies in diagnosing autoimmune neuropathy or in guiding treatment. NF155 is localized to paranodes^9^ and microvilli of myelinating Schwann cells.^10^ NF186 and NF140 are localized to the axolemma mature and immature nodes, respectively. All are produced from the same neurofascin gene and share some subunits (figure 1A).^7,11^ We have therefore tested the frequency, isoform specificity, and clinical correlates of neurofascin antibodies in autoimmune, genetic, and idiopathic neuropathy cohorts.

**Figure 1:**
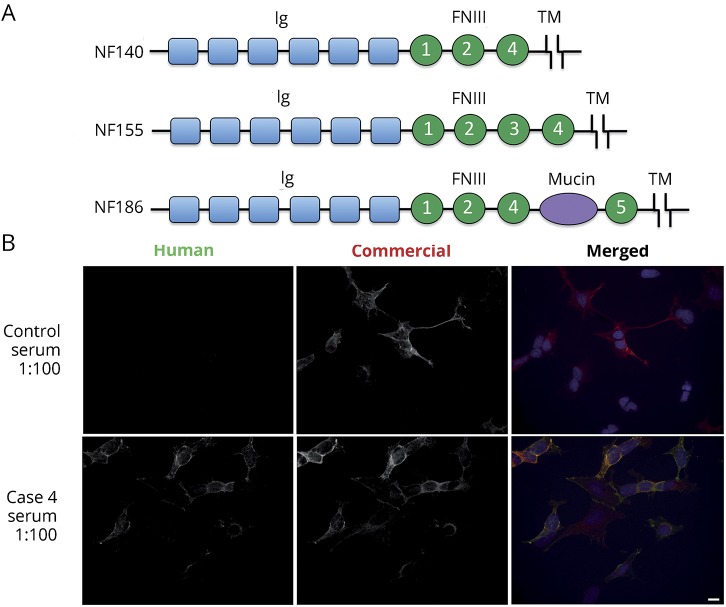
Neurofascin (NF) isoforms and cell-based assay (A) NF155, NF186, and NF140 are isoforms of neurofascin that share common extracellular domains (immunoglobulin [Ig], fibronectin [FN], transmembrane [TM], and mucin), as illustrated (adapted from Zhang et al.^11^ with permission). (B) HEK293 cells were transfected to express either NF155 or NF186, immunolabeled live with sera, fixed, permeabilized, and immunolabeled with a commercial neurofascin antibody, followed by appropriate secondary fluorescent antibodies. This example shows human IgG staining of cells transfected to express NF155 for a patient with chronic inflammatory demyelinating polyneuropathy (case 4) and a control. Scale = 10 μmol/L.

## Methods

### Standard protocol approvals, registrations, and patient consents

Cases with GBS, CIDP, idiopathic neuropathy, and Charcot-Marie-Tooth (CMT) neuropathy were identified from our tissue bank at the University of Pennsylvania (Institutional Review Board protocol 816805). An additional 76 cases with a genetically confirmed diagnosis of CMT were identified at the National Hospital for Neurology and Neurosurgery, Queen Square Hospital, London, as approved by the National Hospital for Neurology and Neurosurgery Research Ethics Committee/Central London (REC 3 09/H0716/61). Of the 76 patients (39 male and 37 female patients, mean age 46 years), 49 had demyelinating CMT and 27 had axonal CMT of the following subtypes: CMT1A (n = 37), CMT1B (n = 1), CMT1C (n = 1), CMT2A (n = 1), CMT2F (n = 5), CMT4B (n = 1), CMTX1 (n = 12), and hereditary sensory neuropathy due to *SPTLC1* (n = 20) and *SPTLC2* (n = 1) mutations.

Data analysis was conducted under Institutional Review Board protocol 820981 at the University of Pennsylvania.

### Neurofascin antibody detection and characterization

Ad293 cells (Agilent Technologies, Santa Clara, CA) were plated on poly-d-lysine–coated coverslips (Neuvitro, Vancouver, WA) in fetal bovine serum–supplemented Dulbecco modified Eagle culture media (DMEM) and allowed to incubate for 24 hours. Cells were transfected with the pFLAG-CMV-5a,b,c expression vector containing mouse NF186 or the pcDNA3 expression vector containing rat NF155 (both courtesy of Dr. Peter Brophy, University of Edinburgh). Samples reactive against NF155 or NF186 were also tested against mouse NF140 with a plasmid also generated by Dr. Brophy.^11^ Cells were transiently transfected with the Jetprime Polyplus Transfection Reagent (Polyplus-transfection SA, Illkirch-Graffenstaden, France). Four hours after transfection, the culture media was replaced with fresh DMEM, and cells were then allowed to incubate for another 16 to 20 hours.

Cells were live-stained with patient sera diluted (1:100) in DMEM for 30 minutes at 37°C, fixed with 4% paraformaldehyde, permeabilized with 0.3% Triton X-100 (Sigma Aldrich, St. Louis, MO), and blocked with 5% normal goat serum in PBS for 1 hour at room temperature before incubation with Alexa Fluor 488–conjugated goat anti-human IgG (H + L) (1:2,000) or IgG Fc_γ_ (1:2,000) antibodies (Jackson ImmunoResearch, West Grove, PA) or with Alexa Fluor 488–conjugated goat anti-human IgM antibodies (1:4,000) (Life Technologies, Carlsbad, CA) for 40 minutes at room temperature. Cells were then treated for 1 hour with polyclonal chicken anti-neurofascin primary antibodies (R&D Systems, Minneapolis, MN), followed by Alexa Fluor 594–conjugated goat anti-chicken IgY (H + L) antibodies for 40 minutes (1:2,000). Cells were washed with PBS between incubations. Cells were mounted with DAPI Fluromount-G (Southern Biotech, Birmingham, AL). Staining results were visualized with a Leica fluorescent microscope.

### IgG subclasses

IgG subtypes were determined by a live staining protocol. After blocking in 5% normal goat serum in PBS, cells were labeled with ThermoFisher unconjugated mouse antibodies against IgG1 (1:200), IgG2 (1:1,000), IgG3 (1:500), or IgG4 (1:75) for 1 hour at room temperature, followed by Alexa Fluor 488–conjugated goat anti-mouse IgG (H + L) antibodies (1:2,000) (Jackson ImmunoResearch) for 40 minutes. Cells were then washed and stained with anti-neurofascin antibodies as described above.

### Statistics

Frequencies of the antibodies in the different groups were compared with the Fisher exact test.

## Results

### Neurofascin antibody frequency

We screened sera from our cohorts of patients for IgG and IgM autoantibodies to NF155 and NF186 using cell-based assays (examples shown in figure 1B). Neurofascin antibodies were more common in patients with GBS/CIDP (14%, 8 of 59) compared to controls with genetic neuropathy (3%, 3 of 111, *p* = 0.01). Patients with idiopathic neuropathy had intermediate frequency of the antibodies (7%, 3 of 43, not significantly different from other groups). The characteristics of antibody-positive patients with GBS and CIDP are presented in table 1.

**Table 1:**
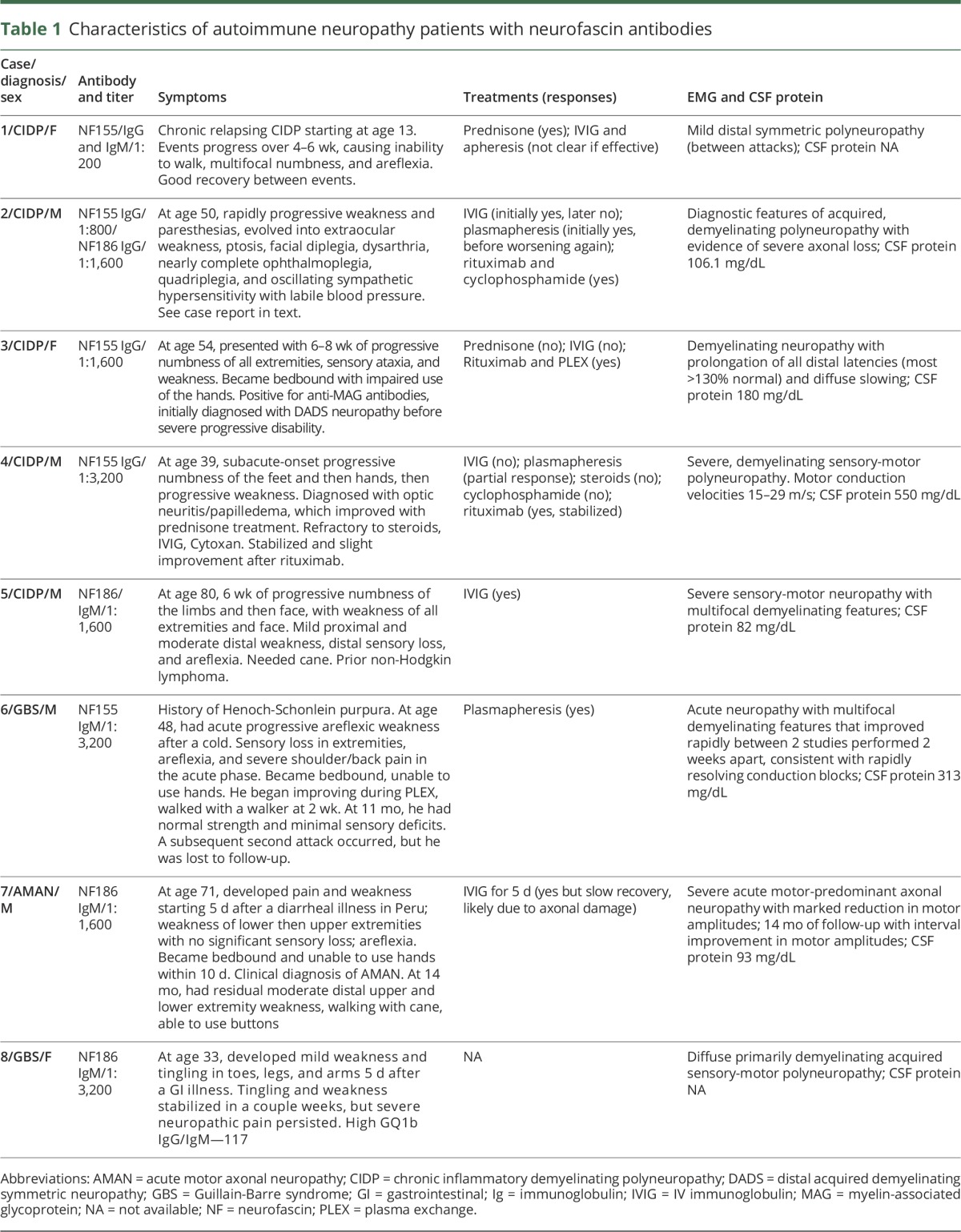
Characteristics of autoimmune neuropathy patients with neurofascin antibodies

### IgG responses to NF155

We detected NF155 IgG binding in 4 of 40 (10%) patients with CIDP, 0 of 14 (0%) patients with GBS, and 1 of 111 (1%) patients with CMT, indicating a low false-positive rate. None (0 of 43) of our idiopathic neuropathy patients had NF155 IgG.

The case with CMT was a 34-year-old man with a c.374T>C (p.Leu125Pro) mutation in *LITAF* that was deemed to be pathogenic because of its absence in control databases, segregation with the disease in other affected family members, and the large physicochemical difference between leucine and proline.

The sera with NF155 IgG were studied with subtype-specific secondary antibodies for IgG1, IgG2, IgG3, and/or IgG4 (table 2). Our patients with CIDP with IgG to N155 all had IgG4-predominant responses, consistent with prior reports.^2,8^ The single patient with CMT1 and NF155 IgG had a high titer (1:3,200) that was predominantly IgG1; no IgG2 through IgG4 antibodies were detected.

**Table 2:**
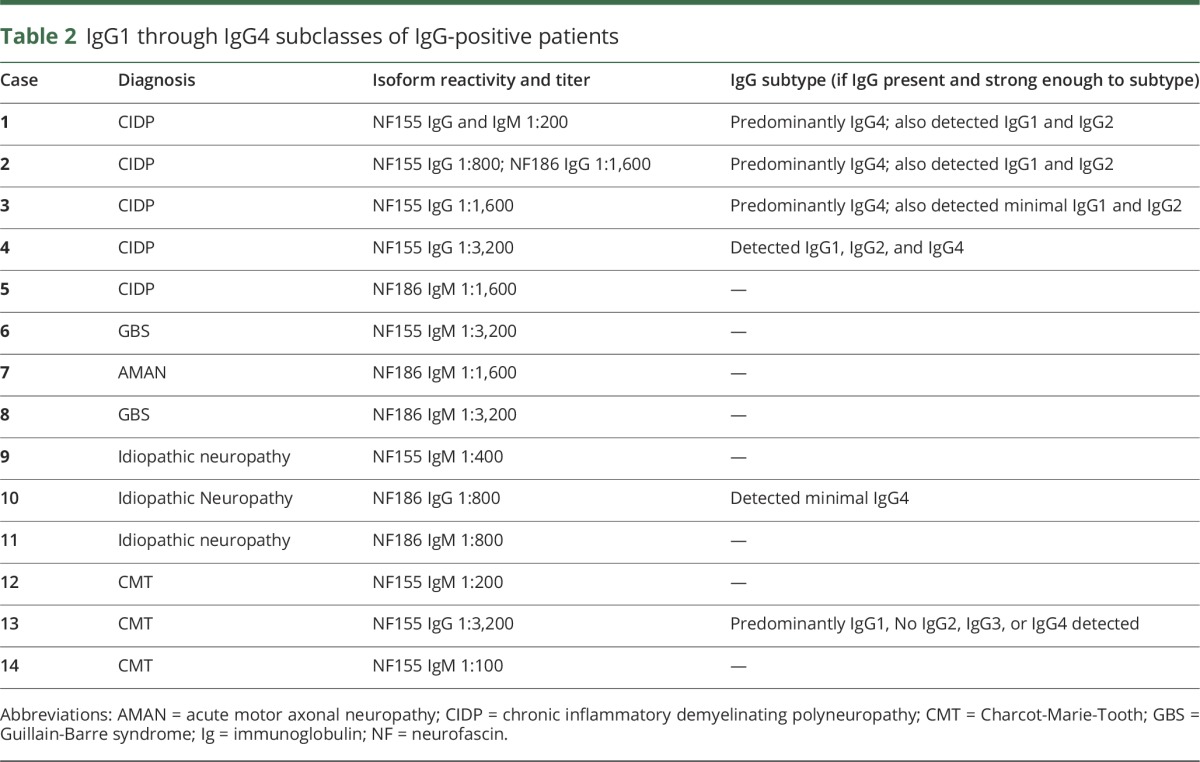
IgG1 through IgG4 subclasses of IgG-positive patients

Our patients with CIDP with NF155 IgG antibodies share important features with previously reported patients.^2^ Specifically, 3 patients had high CSF protein; severe, progressive CIDP; and poor response to IVIG treatment. After rituximab, 2 of the 3 patients showed marked improvement, and the third patient appears to be responding 3 months after treatment. None of them showed disabling tremor, in contrast to prior reports. Case 3 (table 1) had coexisting NF155 IgG and anti–myelin-associated glycoprotein antibodies (with IgMκ paraprotein), although her clinical course was similar to that of the other patients with NF155 IgG. Specifically, she had progressive distal and proximal weakness with loss of ability to ambulate over several months and then rapidly improved after rituximab, a pattern more typical of NF155-positive patients than patients with distal acquired demyelinating symmetric neuropathy.^12^

Case 2 had a more severe phenotype than previously reported and IgG4 recognizing NF140, NF155, and NF186. His clinical and immunologic characteristics are detailed below.

### Case report of a patient with IgG4 to all forms of neurofascin

Starting 1 to 2 weeks after a diarrheal illness, a 50-year-old man developed 2 weeks of progressive ascending weakness and paresthesias. On presentation, his examination was notable for proximal and distal weakness and areflexia. A lumbar puncture demonstrated mildly elevated protein (60 mg/dL) but was otherwise unremarkable. He received 2 g/kg IVIG over 5 days with dramatic improvement and was discharged home 1 day after completing treatment.

Within 3 to 4 days of discharge, the ascending weakness and paresthesias returned. On examination, he was areflexic and had decreased sensation of cold, pinprick, and light touch distally in all extremities. His strength was 2/5 to 4/5 (Medical Research Council scale) in all upper extremity muscle groups bilaterally, weaker proximally. Repeat CSF protein was 106 mg/dL. EMG with nerve conduction studies showed prolonged tibial and peroneal F waves and preserved sensory responses with severely reduced recruitment in multiple proximal limb muscles. He did not meet electrodiagnostic criteria for CIDP.^13^ His GQ1b, HIV, and Lyme antibodies, skeletal survey, and serum immunofixation were normal.

He was again treated with IVIG (2 g/kg total) but continued to decline; he had limited extraocular muscle motility, ptosis, facial diplegia, dysarthria, and 0/5 limb strength throughout with the exception of 2/5 in finger movements and 3/5 dorsiflexion. Repeat EMG with nerve conduction studies showed prolonged distal motor latencies and prolonged F waves diagnostic of CIDP and absent sensory responses. He was treated with a third course of IVIG. One week after transfer, he was intubated for increased secretions and worsening respiratory status. At that time, he had nearly complete ophthalmoplegia, weak cough and gag, 1/5 neck flexion, and quadriplegia. He also had oscillating sympathetic hypersensitivity with labile blood pressure.

Plasmapheresis and IV solumedrol were initiated, with transient improvement. His facial strength improved to the point that lip reading was possible, and he recovered some eye movement. His ptosis improved, and he was able to lift his head off the bed. On day 36, he recovered the ability to wiggle his fingers and toes, but he soon worsened. Cyclophosphamide (750 mg/m^2^) was initiated on day 52 for the first of 3 monthly doses. At this point, he was communicating by protruding his tongue for “yes.” A second course of IV solumedrol and a second round of plasmapheresis were initiated, and his examination improved. He was able to open his eyes, to move his eyes in all directions, and to move his lips to mouth words. However, he developed aspiration pneumonia, and he again became essentially “locked in,” unable to move. A third round of plasmapheresis was initiated, and he received his second dose of monthly cyclophosphamide. On day 86, he received his first of 3 weekly doses of rituximab (375 mg/m^2^). On day 104, his ophthalmoparesis began improving, and he was able to move his jaw. Over the course of the next 2 weeks, he was able to open his eyes, stick out his tongue, and nod his head. He was transferred to a long-term ventilator facility.

Ten months after his initial presentation, he was transferred to acute rehabilitation, decannulated, and gradually advanced to a normal diet. At the last follow-up 19 months after symptom onset, he was able to ambulate with bilateral ankle-foot orthotics, drive, and grip utensils. There was still severe weakness of all ankle and foot movements, as well as moderate weakness of intrinsic hand muscles.

### IgG responses to NF186

In addition to case 2, a low-titer NF186 IgG response (without coexisting NF155 reactivity) was detected in 1 patient with idiopathic neuropathy. No other cases of CIDP, GBS (0 of 14), or CMT (0 of 111) were associated with isolated reactivity to NF186.

### IgM responses to NF155 or NF186

IgM responses to NF155 or NF186 were detected in 5 of 59 (8%) patients with autoimmune neuropathy: 2 with CIDP (cases 1 and 5), 1 with acute inflammatory demyelinating polyneuropathy (case 6), 1 with acute motor axonal neuropathy (case 7), and 1 with features of CIDP and GBS (case 8). One patient with CIDP (case 1) had IgM against NF155 with a coexisting IgG4 response to NF155. IgM responses to NF186 or NF155 were also detected in 2 of 111 (2%) patients with CMT and 2 of 43 (5%) patients with idiopathic neuropathy. The 2 cases with CMT included a 78-year-old man with CMT1A and a relatively mild phenotype as defined by a CMT examination score of 7 and CMT neuropathy score of 12 and a patient with progressive neuropathy from age 3 without a definitely identified mutation.

### Epitope mapping of neurofascin IgG responses

**Figure 2:**
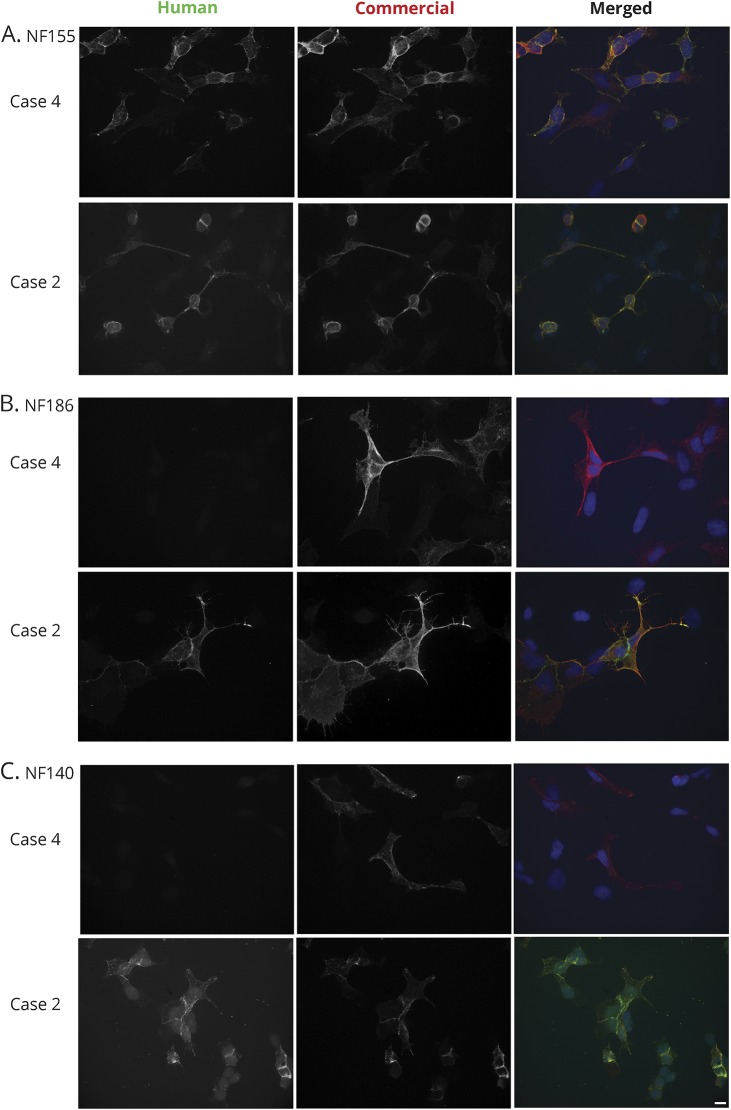
Isoform specificity of neurofascin (NF) responses HEK293 cells were transfected to express NF155 (A), NF186 (B), or NF140 (C), immunolabeled live with sera, fixed, permeabilized, and immunolabeled with a commercial neurofascin antibody, followed by appropriate secondary fluorescent antibodies. Most cases with neurofascin antibodies, like case 4, were specific to NF155 or NF186. Case 2 is so far unique in recognizing all 3 isoforms. See text. Scale 10 μmol/L.

NF155, NF186, and NF140 share some subunits in their extracellular domains (figure 1A). To determine the target epitopes of our NF155 or NF186 IgG–positive samples, we used cell-based assays for each of the 3 isoforms (figure 2). Isoform specificity and titers are summarized in table 2. Three of 4 sera with NF155 IgG responses did not recognize NF186 or NF140. These patients most likely had antibodies against epitopes involving the third fibronectin-like domain, which is the only domain expressed in NF155 that is not present in both NF186 and NF140. Case 2, however, had strong IgG reactivity against all 3 neurofascin isoforms, indicating that at least 1 target epitope was in a domain that was shared by all 3 neurofascin isoforms. Because all of the domains of NF140 are also present in NF155 and NF186, a response to NF140 is sufficient to explain his response to NF155 and NF186, but this does not exclude the possibility that his antibodies also recognize domains specific to NF155 or NF186.

### Persistence of responses over time

When possible, we analyzed follow-up samples from CIDP and neurofascin reactivity. Follow-up samples from 2 patients with NF155 IgG were studied. Case 2 provided samples during initial onset, 2 months later (during severe symptoms), and 19 months later, after the patient had significant recovery. The first 2 samples showed strong NF140, NF155, and NF186 IgG reactivity, whereas the final sample was negative for all neurofascin isoforms. In contrast, case 1, who had relapsing/remitting CIDP, had 2 samples collected 2 years apart, both of which were positive (she had had no attacks in years).

Follow-up samples were also obtained for 3 patients with IgM responses but no IgG responses. Case 5, who had CIDP and responded to IVIG treatment, continued to have IgM antibodies against NF186 over the course of 8 months, during which time 4 samples were collected at 2- to 4-month intervals and the patient was still experiencing weakness but much improved symptoms as a result of regular IVIG infusions. Cases 6 and 7 had GBS and acute motor axonal neuropathy, respectively. Case 6 had IgM against NF155, and case 7 had IgM against NF186 during acute illness. Follow-up samples, after acute symptoms had subsided, were negative for IgM or IgG antibodies. Case 6 subsequently experienced a relapse of his neuropathy but was lost to follow-up.

## Discussion

We comprehensively examined the frequency, clinical correlates, isoform specificity, and persistence of neurofascin antibodies in patients with diverse kinds of neuropathy. Our study supports the specific association of NF155 IgG4 with severe CIDP refractory to IVIG but often responsive to rituximab. These responses were specific to the NF155 isoform for most cases. Transient responses, often IgM, occurred in some patients with GBS. In addition, we found a higher frequency of neurofascin responses in idiopathic neuropathy compared to genetic neuropathy. Finally, we have reported a patient with incredibly severe but treatable neuropathy associated with autoimmunity to the common domains shared by all neurofascin isoforms.

Neurofascin antibodies were first reported in the context of multiple sclerosis^14^ and later in GBS.^15^ Subsequent reports have described NF155 and/or NF186 IgM or IgG responses in 1% to 18% of patients with GBS and CIDP, depending on the diagnosis and type of antibody.^1^ An association of NF155 IgG with CIDP particularly became clearer in subsequent reports,^4^ followed by the association of NF155 IgG4 with CIDP resistant to IVIG treatment and responsive to rituximab in small groups of patients.^2,8,16^ Most recently, a study of 246 patients with CIDP found 5 patients (2%) with IgG4-predominant responses to the common epitopes of NF140, NF186, and NF155.^17^ These patients generally had subacute onset, prominent sensory ataxia, and sometimes cranial nerve involvement. While these patients had some similarities to case 2, none were as severely affected. As with our case, clinical remission correlated with resolution of the antibody response.

NF155 IgG4 was found in 10% of our patients with CIDP, so screening appropriate patients may be clinically useful. One case had coexisting myelin-associated glycoprotein antibodies and met criteria for distal acquired demyelinating symmetric neuropathy but was otherwise similar to our other NF155 IgG-positive cases.

Transient IgM responses to neurofascin occurred in some of our patients with GBS, and some of our patients with NF155 IgG4 initially presented with an acute-onset neuropathy resembling GBS. Because immunoglobulins are thought to be progressively expressed by maturing B cells in a specific order (IgM→IgG3→IgG1→IgG2→IgG4),^18^ it is likely that patients who have an IgG4 against NF155 started with an IgM against NF155. IgM responses to NF155 or NF186 were, however, found in several different groups of patients (those with idiopathic neuropathy, CMT, CIDP, and GBS), so they do not invariably progress to IgG4 responses. Additional studies are needed to determine whether patients with GBS with neurofascin IgM are at higher risk of progressing to CIDP or whether IgM antibodies contribute to pathogenesis.

In most cases, antibody responses were specific to either NF155 or NF186, consistent with prior studies showing that NF155 antibodies recognize the third and fourth fibronectin-like domains^4^ and that most NF155 antibodies recognize a glycosylated epitope in the first through fourth fibronectin-like domains.^19^ Case 2 had reactivity to an epitope shared by all 3 isoforms. This “pan-neurofascin” immune response could potentially affect both NF186 (localized to nodes) and NF155 (mostly localized to paranodes) and may account for the severe phenotype observed. Other patients with CIDP and antibodies to NF155 have been disabled and unable to ambulate, but the nearly locked-in state of our patient is unprecedented. It would be informative to study other patients with extremely severe CIDP for this antibody response. Additional studies may determine whether the disappearance of the neurofascin antibody response, as in this case, is a reliable biomarker of clinical CIDP remission. Even in the setting of profound disability, immune therapy and patience may lead to a significant recovery.

The NF155 IgG responses we observed were IgG4 predominant, matching prior reports. IgG4 antibodies have unique properties, including being functionally monovalent as a result of hemi-antibody exchange, having very high affinities, and not fixing complement.^20^ IgG4 antibodies to CAMs like neurofascins may act by disrupting their interactions with other CAMs.^21^ Testing for IgG4 to NF155, not just IgG to NF155, may increase the specificity of a positive finding for treatment-resistant CIDP.

By some estimates, almost half of patients with chronic progressive neuropathy are idiopathic, having no identifiable cause.^7,22^ It is possible that some patients with idiopathic neuropathy may have an autoimmune cause even though they lack the distinctive clinical and electrophysiologic findings of GBS or CIDP. The possibility of an increased frequency of neurofascin antibodies in idiopathic compared to genetic neuropathy therefore requires further investigation.

Our work supports the concept that autoantibodies to peripheral nerve proteins such as neurofascins may define particular phenotypes and predict response to treatment. Our work also demonstrates that different types of antibody responses to the same antigen (IgM vs IgG, persistent vs transient) or antibody responses to different parts of the same protein (NF155-specific domains vs core neurofascin domains) have different prognostic implications.

## Glossary

CAM: cell adhesion molecule
CIDP: chronic inflammatory demyelinating polyneuropathy
CMT: Charcot-Marie-Tooth
DMEM: Dulbecco modified Eagle culture media
GBS: Guillain-Barre syndrome
IG: immunoglobulin
IVIG: IV immunoglobulin
NF: neurofascin
